# Impact of data protection regulation on Slovenian eHealth

**DOI:** 10.7189/jogh.11.03063

**Published:** 2021-03-27

**Authors:** Lucija Tepej Jočić

**Affiliations:** National Institute of Public Health, Ljubljana, Slovenia

The paper elaborates the impact of personal data protection regulation on the Slovenian eHealth system [1,2], in particular the Central Registry of Patient Data (CRPD). Slovenia has had restrictive data protection regulations since 2004 [3], resulting in an elaborate legislative and governance framework for data processing in health care.

The Central Registry of Patient Data (CRPD) is a core platform of the Slovenian eHealth system, enabling sharing of electronic health records on a national level. It is comprised of many diverse types of unstructured medical documents and patient summary records. Currently, CRPD contains over 50 million records, covering over 90% of the population.

## LEGISLATIVE FRAMEWORK

According to the Constitution of the Republic of Slovenia, personal data protection is deemed a constitutional right. Accordingly, Slovenia has implemented the highest standards of personal data protection. The Personal Data Protection Act [3] entered into force in 2004. Not only are the stipulations in line with GDPR, the Personal Data Protection Act outperforms GDPR in terms of restrictions in public health care. As a result, GDPR did not bring much change to Slovenian health care. It had, however, escalated concerns due to the threat of enormous fines, potentially devastating for the ever-underfunded health sector. Public sector entities are only permitted to process personal data if a specific law allows this explicitly. This stipulation has a crucial impact on health data processing, as most of Slovenian health care providers are public entities. Sectorial regulations specify databases in full detail, including content, purpose, scope, data subjects, users and linkage of data sources. Consequently, legislative procedure is needed even for minor changes of existing databases, let alone introduction of new ones.

According to the Healthcare Databases Act [4], the National Institute of Public Health is the lawful controller of CRPD. No patient consent is needed for data processing, and all health care providers are eligible users. The Healthcare Databases Act specifies the content specifically. There is a broad definition of health care documentation comprised of any material related to health care treatment. As opposed to the health care documentation, an explicit list of Patient Summary sections is defined, identifying demographic and health related data, such as allergies, diseases, medical procedures, vaccinations, implants and medication history.

Access rights are stipulated in the subordinate Rules on authorizations for data processing in the Central Registry of Patients Data [5]. Only the chosen personal doctor has unlimited access. Healthcare documentation is only accessible to medical doctors, based on the patient's choice of personal doctor, an active referral or appointment, patient consent or emergency access. It is further restricted with regard to medical practice settings; the practice setting of the issuing institution must comply with the practice setting of the inquiring user. As opposed to health care documentation, the Patient Summary is widely accessible to all health care professionals unless the patient has prohibited access explicitly, as defined in the Rules on the prohibition of access to the patent’s data in CRPD [6].

## TECHNICAL IMPLEMENTATION OF LEGISLATIVE FRAMEWORK IN CRPD

Healthcare providers are connected to CRPD via a proprietary interface called the IH Adapter. The IH Adapter is based on the IHE XDS [7] and OpenEHR standards [8]. Applications used by health care providers need to be adjusted accordingly. The IH Adapter comprises organisational and legislative specifics of the Slovenian health system. As for legislative specifics, complex algorithmic access rules are implemented.

End users are registered in the eHealth Users Database with digital certificates. The eHealth user database is linked to the Registry of health care providers and health care professionals. In the Registry, an individual doctor has a practice setting code assigned by their employer, aligned to their medical speciality and actual employment.

Access authorizations are based on personal doctor assignment, a valid referral, eventual patient’s consent or emergency service. Full access is only granted if the querying doctor’s ID matches the pre-registered ID of the chosen doctor in the Patient Registry. For other doctors, the patient’s active referrals to the health care provider are verified, and access is only granted if a valid referral exists within the given time.

Eventual patient consent must be duly pre-recorded on site. Upon the patient’s explicit statement, the doctor submits the opt-in policy document via their local medical record processing application and thus obtains access without being the chosen doctor or having an active referral. A similar procedure applies for emergency access. Upon submission of the emergency access note, the aforementioned restrictions can be temporarily bypassed for a maximum duration of 8 hours.

Queries to CRPD are only enabled for end users by means of a personal digital certificate, and an authentication token containing mandatory attributes; health care provider, user role and practice setting. Upon registration with a personal digital certificate, the attributes of the authorization token are evaluated regarding professional qualification and medical practice setting to which the doctor is entitled by their employer. Every single query is the subject of a multi factorial auditing process embedded in the CRPD application. The health care professional’s ID is checked against the assignment of the chosen doctor in the Patient Index. Current referrals and appointments are checked against the health care provider identifier. The eventual patient’s explicit consent and Break the Glass recordings are checked. If neither of the aforementioned conditions are met, the doctor is only granted access to the Patient Summary. Otherwise, the system evaluates metadata of the available documents with regard to practice settings of the querying doctor. The resulting list of available documents is filtered against the lawful matrix combining practice setting codes of the querying doctor and metadata of the patient’s documents.

**Figure:**
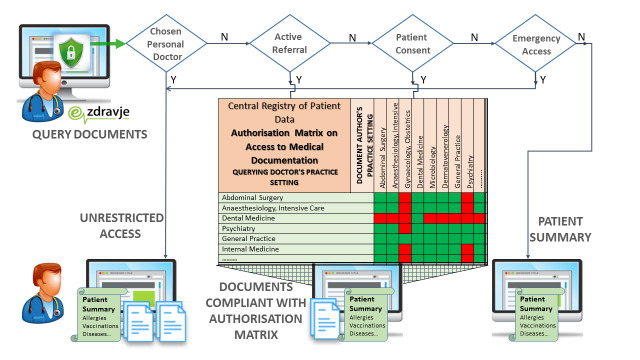
Photo: Rules on authorizations for data processing in the Central Registry of Patients Data (created by the author, used with permission).

Accordingly, every single query is subject to elaborate real time evaluation, and the criteria attributes are stored in four databases, namely the CRPD Patient Directory, the CRPD IHE XDS Document System [7], the CRPD OpenEHR Database [8], and the eHealth Users Database. The aforementioned process requires minimal implementation in the hospital information system. The hospital information system only needs to initiate registration and pass the authentication token to central service. This grants National Institute of Public Health full control of end user access and enables them to exercise the role of data controller irrespective of the diversity of information systems used by health care providers and irrespective of the local applications in use. The implementation of CRPD is considered a model example of default and embedded data protection.

## LESSONS LEARNT

CRPD has been in operation since 2015. Ever since, most of the stakeholder feedback has concerned authorizations of health care professionals.

Data availability complaints are by far the most common issue reported by end users, representing up to 80% of questions and fault reports. As substitutions are common in primary care, patients are often treated by doctors other than the chosen one, and substitutive doctors have no default access. Data in the Healthcare Providers Registry is often incomplete and doctors are denied access due to missing assignment of practice setting codes. Some referrals are not duly recorded due to technical errors, and medical specialists are denied lawful access. These issues can be bypassed with a patient’s consent, but on-site consent management is an unwelcome administrative burden to health care professionals.

A substantial amount of resources is needed to maintain existing access mechanisms as well as to upgrade them upon introduction of new content. Provided that human and financial resources for eHealth are severely constrained, the capacity for further development is reduced.

Exhaustive legislation did not help to resolve uncertainty, and health care professionals find them difficult to understand. Meticulous definitions do not result in unanimous interpretations, and rights and obligations regarding data processing are questioned. eHealth authority often needs to consult the data protection authority on data controllership matters, especially when introducing new services.

Embedded access rules have neither consolidated conflicting views of medical specialities, nor have they ensured trust among eHealth stakeholders. Albeit maximal restrictions apply for psychiatric documents, psychiatrists remained reserved, and the university psychiatric clinic still refuses to participate. Similarly, some microbiology laboratories abstain from submitting certain allegedly stigmatising results. Despite the fact that nurses are actively involved in primary care and emergency treatment, some doctors believe that they are not eligible to access Patient Summaries. Although these kinds of sceptical opinions are relatively rare, they are supported by influential individuals.

Constraints of legal ground for health data have been evident on numerous occasions. Given that every database needs to be defined by law, legal procedure must be initiated not only for every new database but also for minor modifications, such as adding a new data element. The procedure of amending a law is demanding and typically lasts several years. Subordinate regulation is written by the Ministry of Health, but this actually depends on political priorities, and eHealth is a low priority.

The COVID-19 pandemic has shed a harsh light on the above mentioned issues. Restrictive access policies have been proven to be too rigid for the crisis mode of operation. Microbiology reports are banned for dental medicine, and dentists protested for being deprived of COVID results. Provided that nurses have an active role in triage and admission procedures, the Patient Summary was proposed as a handy source of COVID status. However, leading epidemiologists and microbiologists believed disclosing such a sensitive information would increase the risk of unauthorised use, leakage, stigmatising infected patients and even denial of health care service. The pandemic has indeed severely impeded access to health care, and some patients have deliberately denied their COVID status in hope for faster admission. Routine testing was not widely available, health care associated infections were common and denying patients were increasing the risk of onward transmissions. As the situation worsened, numerous complaints were expressed by health care professionals and formal demands were addressed to authorities for unconstrained access to COVID status. National consensus on adding COVID status to the Patient Summary was not reached until the devastating second wave of the epidemic in November 2020.

## CONCLUDING REMARKS

Balancing data protection and data accessibility is one of the greatest challenges of digital health. On the one hand, a more open access ensures data availability in unpredictable situations but provokes privacy objections. On the other, harsh restrictions jeopardise flawless data exchange. The Slovenian Central Registry of Patient Data is a model example of privacy by design. However, the governance of such a complex system is challenging and resource consuming. Implementation and administration issues are emerging, impairing data availability and usability. Since legislative procedure is not able to follow the evolution of the health care system and underlying digital technologies, the current regulatory framework is supporting neither the optimal implementation of eHealth services nor their future development.

The future digitalisation of Slovenian health care would be facilitated by a profound change of legal base. The law on health data processing could only lay out the general stipulations whereas the detailed descriptions of databases could better be defined by a more flexible subordinate regulation. A partial solution would be to simplify the existing Rules on Access by focusing solely on medical qualifications and disregarding the circumstantial factors such as referrals and pre-election of a personal doctor. This approach would grant equal access to all doctors and let them take full responsibility to autonomously select the documents they deem relevant for treatment. Provided that audit trail exists for every single transaction, suspected violations of privacy rights could be investigated in retrospect. In the end, it is the doctor, and not the system, who is authorised to decide on the relevant information. A rigorous system may accidently restrain relevant data, and the deprivation of relevant data may deteriorate the quality of medical service. If the ultimate goal of digitalization is improvement of health care, too rigorous data protection mechanisms may actually imperil this ultimate goal.
